# Risk factors for bloodstream infection in patients at a Brazilian hemodialysis center: a case–control study

**DOI:** 10.1186/s12879-015-0907-y

**Published:** 2015-03-26

**Authors:** Dayana Fram, Meiry Fernanda Pinto Okuno, Mônica Taminato, Vinicius Ponzio, Silvia Regina Manfredi, Cibele Grothe, Angélica Belasco, Ricardo Sesso, Dulce Barbosa

**Affiliations:** School of Nursing, Paulista School of Nursing, Universidade Federal de São Paulo (Federal University of São Paulo – EPE/UNIFESP), R. Napoleão de Barros 754, São Paulo, 04024-002 Brazil; Infection Control Unit, Children’s Institute and Institute for the Treatment of Childhood Cancer, School of Medicine, Universidade de São Paulo (University of São Paulo – ITACI/FMUSP), Av. Dr. Enéas Carvalho de Aguiar 647, São Paulo, 05403-000 Brazil; Division of Infectious Diseases, Escola Paulista de Medicina, Universidade Federal de São Paulo (Paulista School of Medicine, Federal University of São Paulo – EPM/UNIFESP), R. Napoleão de Barros, 715, 7° andar, São Paulo, 04024-002 Brazil; Division of Dialysis, Hospital do Rim e Hipertensão, Fundação Oswaldo Ramos, (Kidney and Hypertension Hospital, Foundation Oswaldo Ramos - HRIM/FOR), R. Pedro de Toledo 282, São Paulo, 04039-030 Brazil; Division of Nephrology, Paulista School of Medicine, Universidade Federal de São Paulo (Federal University of São Paulo – EPM/UNIFESP), R. Botucatu 740, São Paulo, 04023-900 Brazil

**Keywords:** Dialysis, Bacteremia, Risk factors, *Staphylococcus aureus*

## Abstract

**Background:**

Infection is the leading cause of morbidity and the second leading cause of mortality in patients on renal replacement therapy. The rates of bloodstream infection in hemodialysis patients vary according to the type of venous access used. Gram-positive bacteria are most frequently isolated in blood cultures of hemodialysis patients. This study evaluated risk factors for the development of bloodstream infections in patients undergoing hemodialysis.

**Methods:**

Risk factors associated with bloodstream infections in patients on hemodialysis were investigated using a case–control study conducted between January 2010 and June 2013. Chronic renal disease patients on hemodialysis who presented with positive blood cultures during the study were considered as cases. Controls were hemodialysis patients from the same institution who did not present with positive blood cultures during the study period. Data were collected from medical records. Logistic regression was used for statistical analysis.

**Results:**

There were 162 patients included in the study (81 cases and 81 controls). Gram-positive bacteria were isolated with the highest frequency (72%). In initial logistic regression analysis, variables were hypertension, peritoneal dialysis with previous treatment, type and time of current venous access, type of previous venous access, previous use of antimicrobials, and previous hospitalization related to bloodstream infections. Multiple regression analysis showed that the patients who had a central venous catheter had an 11.2-fold (CI 95%: 5.17–24.29) increased chance of developing bloodstream infections compared with patients who had an arteriovenous fistula for vascular access. Previous hospitalization increased the chance of developing bloodstream infections 6.6-fold (CI 95%: 1.9–23.09).

**Conclusions:**

Infection prevention measures for bloodstream infections related to central venous catheter use should be intensified, as well as judicious use of this route for vascular access for hemodialysis. Reducing exposure to the hospital environment through admission could contribute to a reduction in bloodstream infections in this population.

## Background

Infection is the leading cause of morbidity and the second leading cause of mortality among patients on renal replacement therapy [1]_._ Mortality from all causes in patients on dialysis treatment is 6.5–7.9 times higher than that of the general population. In patients ≥65 years of age, mortality from infection is two times higher than that in a population of the same age with a diagnosis of diabetes mellitus, neoplasms or congestive heart failure [1]. According to the US Renal Data System [1], the mortality rate at the end of the first year of hemodialysis decreased by 38% for cardiovascular causes and by 50% for infectious causes between 2000 and 2010. Among patients on hemodialysis, the mortality rate decreased by 26% between 1985 and 2000 [1]. When comparing mortality rates between the second and 12th month after initiation of hemodialysis, there was a significant reduction in mortality from all causes, which dropped from 440 to 201 deaths per 1000 patients/year. The mortality rate from infectious causes decreased from 43 to 19.4 deaths per 1000 patients/year [1].

North American data indicate that bloodstream infection (BSI) rates in patients on hemodialysis vary between 0.5 and 27.1 per 100 patients/month depending on the type of venous access used [2]. More recent studies report a reduction in the incidence of BSI in this population between 1.09–0.89 and 2.04–0.75 per 100 patients/month after implementation of specific control measures [3,4]. This decrease was attributed to national initiatives for the reduction of healthcare-related infections [5].

The literature reports that the use of a central venous catheter (CVC), hypoalbuminemia, diabetes mellitus, anemia, female gender and colonization by *methicillin-resistant Staphylococcus aureus* (MRSA) are risk factors for the development of BSI in hemodialysis patients [6-10]. Gram-positive bacteria are the most frequently isolated agents in blood cultures from hemodialysis patients, mainly *S. aureus* [7,10,11].

According to the US Renal Data System, the principal morbidity event for hemodialysis patients is hospitalization for infections, which showed an increase of 43% between 1993 and 2011. On the other hand, hospitalizations for complications from venous access, showed a decrease of 57% [1].

Fram et al*.* reported that the risk factors for morbidity and mortality among patients with BSI on hemodialysis were age, isolation of *S. aureus*, and isolation of a resistant microorganism [12].

Despite the best efforts to prevent BSI specific to hemodialysis patients, BSI still has a large impact [13-16]. Therefore, the objective of this study was to evaluate risk factors for the development of BSI in patients undergoing a Brazilian hemodialysis center.

## Methods

### Ethical considerations

This study was conducted with the approval of the Committee on Ethics and Research at the Federal University of São Paulo.

### Design, period and location of the study

This was a retrospective, case–control study conducted between January 2010 and June 2013 at the hemodialysis satellite unit at the Kidney and Hypertension Hospital of Foundation Oswaldo Ramos (HRIM/FOR) in the city of São Paulo, Brazil. The HRIM/FOR is a nephrology service, considered to be a national and international reference for teaching, research and care activities. It serves an average of 400 patients in the dialysis program/month (221 in hemodialysis). This institution follows rigorous standards for medical record keeping.

Patients with chronic renal insufficiency in a hemodialysis program, ≥18 years of age, with BSI defined according to the specific criteria of the National Healthcare Safety Network Dialysis Event Surveillance Manual (Centers for Disease Control and Prevention) [17], which defines BSI as any positive blood culture, were considered for enrollment in the study. We considered all positive blood cultures regardless of the site where blood was drawn. For common skin commensal pathogens (*Staphylococcus* coagulase negative including *Staphylococcus epidermidis*), we considered BSI when pathogens were cultured from two or more blood cultures and/or the patient was treated by a physician [17,18]. Blood cultures were processed in accordance with the recommendations of the Clinical and Laboratory Standards Institute [19].

The cases were obtained from a survey of microbiology laboratory records at the institution during the study period. For patients who had more than one positive blood culture, only the first episode was considered for this study. These patients were included in the case group. Hemodialysis patients at the same institution, ≥18 years of age, and without positive blood cultures during the study period were used as controls. Pairing criteria were: age, time on hemodialysis, and diagnosis of diabetes mellitus. The proportion of cases and controls was 1:1.

### Study protocol and data collection

Data collection occurred through review of patients’ medical records. For the cases group, data collection included sociodemographic characteristics, comorbidities, body mass index [20], microorganisms in blood culture, resistance profiles of antimicrobials, and previous treatment for end stage renal disease. The type, location and time of current and previous venous access, clinical complications (transfusions, number of transfusions, hospitalization because of infection, length of hospital stay, prior antimicrobial use, previous hospitalization, and number of previous hospitalizations), occurrence of death, and transfer of treatment were recorded for a period of 6 months prior to the BSI.

We defined short duration CVC as non-tunneled hemodialysis CVC. However some patients remained with this kind of catheter for a longer period due to difficult inserting other more adequate vascular access for hemodialysis. Long duration CVC was defined as tunneled hemodialysis CVC [17]. The time of current and previous venous accesses was stratified by period. This was done as in some situations they may present with similar periods*.*

Prior antimicrobial use was defined as every previous antimicrobial treatment for at least seven days.

Death was considered to be related to BSI when it occurred within 15 days after microbiologic diagnosis of infection [21,22].

Controls were selected from the computerized patient record system, according to the previously mentioned pairings, using the closest date of beginning dialysis in relation to the case group. Of these, the same variables described above were collected, except for the presence of BSI at the beginning of the study.

A microorganism was considered multi-resistant when it showed resistance to a pharmaceutical agent in three or more classes of antimicrobials. *Staphylococcus* was considered multi-resistant when it showed no susceptibility to *methicillin*, and *Enterococcus* was considered multi-resistant when it showed no susceptibility to vancomycin (VRE) [23-25].

### Statistical analysis

A descriptive analysis of the cases and controls was conducted which examined the variables of interest. Data are presented using absolute frequencies and percentages for categorical variables, and mean ± standard deviation for continuous variables. The primary outcome was BSI. Associations between BSI and categorical variables were tested using the chi-square test, Fisher’s exact test or likelihood ratio. The association between the continuous variables and the presence of BSI was examined using the student’s *t*-test or Mann–Whitney test, as appropriate. Univariate logistical regression was used to investigate the relationship between each independent variable (preselected at the crossings where p ≤ 0.05) and the dependent variable, BSI. To compare the outcomes of patients that developed BSI and the patients that did not developed BSI we used only the univariate logistical regression.

Multiple logistic regression analysis was conducted to determine the independent variables that continued to be associated with BSI after inclusion in the model of significant variables in univariate analysis. To include the variables in the regression model, the forward stepwise method was used. Odds ratios were calculated with respective confidence intervals of 95%. SPSS version 19.0 (Chicago, IL, USA) was used for statistical analyses.

## Results

The total number of patients in the dialysis program for the study period was 353, of which 221 (62%) were on hemodialysis and 132 (38%) were on peritoneal dialysis. Ninety-three patients (42.1%) met inclusion criteria. Twelve patients were excluded because they were unable to be paired with a control. The final study population included 162 patients (81 cases and 81 controls). For nine cases, a control of the same age was not available, and for three cases, a control with the same time on hemodialysis was not available.

The sociodemographic characteristics of patients included in the study are presented in Table 1. There was no statistically significant difference in these characteristics among the study population.

**Table 1:** **Sociodemographic characteristics of the cases group and control patients**

	**Case (** ***n*** **= 81)**	**Control (** ***n*** **= 81)**	***p*** **-value**
Age	56 ± 17.1	53 ± 16.3	0.325
**Sex**			
Male	36 (44.5)	47 (58.0)	0.083
Female	45 (55.5)	34 (42.0)	
**Race**			
White	49 (60.5)	55 (68.0)	0.325
Nonwhite	32 (39.5)	26 (32.0)	
**Educational level**			
No education	21 (26.0)	14 (17.0)	0.198
Elementary	28 (34.5)	22 (27.0)	
High School	22 (27.0)	28 (35.0)	
Higher education	10 (12.5)	17 (21.0)	

Values are expressed as mean ± SD or *n* (%).

For clinical data related to dialysis treatment, the following variables were statistically significant (p ≤ 0.05) in the initial analyses and were preselected for logistic regression: associated diseases (hypertension), previous treatment (history of peritoneal dialysis), current type of venous access, duration of current venous access, use of previous venous access, previous type and duration of tunneled hemodialysis CVC, previous use of antimicrobials, and previous hospitalization. Duration of previous venous access had a p-value of ≤0.05; however, it was not included in regression analysis because of its low frequency (Table 2).

**Table 2:** **Clinical variables of interest of the case group and control patients**

				**Case (** ***n*** **= 81)**	**Control (** ***n*** **= 81)**	***p-*** **value**
**Comorbidities**						
Hypertension				71(87.6)	59 (72.8)	0.018
Cardiovascular diseases				17 (20.9)	12 (14.8)	0.306
Diabetes mellitus				26 (32.0)	26(32.0)	1.000
Others*				27 (33.3)	22 (27.2)	0.421
Body mass index, Kg/m^2^				24 ± 4.3	24 ± 3.9	0.945
Previous treatments				56 (69.1)	47 (58.0)	0.141
**Type of previous treatments**						
Conservative				36 (64.3)	30 (37.0)	0.337
Peritoneal dialysis				19 (33.9)	9 (11.1)	0.038
Hemodialysis				17 (30.4)	9 (11.1)	0.087
Transplant				15 (26.8)	8 (9,9)	0.115
Duration of hemodialysis treatment, months				29 ± 39.2	36 ± 42.1	0.276
**Type of current venous access**						
AVF**				25 (30.9)	64 (79.0)	<0.001
Tunneled hemodialysis CVC***				52 (64.2)	13 (16.0)	
Non-tunneled hemodialysis CVC				4 (4.9)	1 (1.2)	
PTFE Graft****				0 (-)	3 (3.7)	
	**Location of current venous access**					
	Upper arm			24 (29.6)	67 (82.7)	
	Jugular vein			50 (61.7)	13 (16.0)	
	Subclavian vein			7 (8.6)	0 (-)	
	Femoral vein			0 (-)	1 (1.2)	
	**Duration of current venous access**					
	0–30			17 (21,0)	3 (3.7)	<0.001
	30–180 days			27 (33.3)	8 (9.9)	
	>180 days			37 (45.7)	70 (86.4)	
Previous venous access (6 months)				26 (32.1)	11 (13.6)	0.005
Number of previous accesses				1.2 ± 0.41	1.3 ± 0.46	0.744
	**Type of previous venous access**					
	AVF			7 (8.6)	3 (3.7)	0.192
	Tunneled hemodialysis CVC			14 (17.3)	5 (6.2)	0.028
	Non-tunneled hemodialysis CVC			5 (6.2)	6 (7.4)	0.755
	PTEF Graft			5 (6.2)	1 (1.2)	0.210
		**Location of tunneled hemodialysis CVC**				
		Jugular vein		13 (16.0)	3 (3.7)	
		Subclavian vein		0 (-)	1 (1.2)	
		**Location of non-tunneled hemodialysis CVC**				
		Jugular vein		4 (4.9)	6 (7.4)	
		Subclavian vein		1 (1.2)	0	
		**Duration of previous venous access**				
		** AVF**				
		0–30		0	1 (1.2)	
		30–180 days		4 (4.9)	0 (-)	
		>180 days		3 (3.7)	2 (2.5)	
		**Tunneled hemodialysis CVC**				
		0–30		4 (4.9)	0 (-)	0.034
		30–180 days		6 (7.4)	5 (6.2)	
		>180 days		4 (4.9)	0 (-)	
		**Non-tunneled hemodialysis CVC**				
		0–30		1 (1.2)	6 (7.4)	
		30–180 days		3 (3.7)	0	
		>180 days		1 (1.2)	0	
		PTFE Graft**				
		0–30		0 (-)	0 (-)	
		30–180 days		1 (1.2)	0 (-)	
		>180 days		4 (4.9)	1 (1.2)	
Transfusions				11 (13.6)	7 (8.6)	0.317
Number of transfusions				1.9 ± 2.7	1 (-)	0.246
Hospitalizations due to infection				21 (25.9)		
Length of hospital stay				16.9 ± 16.4		
Previous use of antimicrobials				28 (34.6)	14 (17.3)	0.012
Previous hospitalizations				19 (23.5)	4 (4.9)	<0.001
Number of previous hospitalizations				1.5 ± 1.2	1(-)	0.259

Values are expressed as mean ± SD or *n* (%). *hypothyroidism, hyperthyroidism, neoplasm and s*ystemic lupus erythematosus*; **AVF: arteriovenous fistula; ***CVC: central venous catheter; ****PTFE, polytetrafluoroethylene.

Table 3 shows the results of logistic regression analysis. In univariate analysis, variables significantly associated with the occurrence of BSI were: hypertension, history of peritoneal dialysis, type and duration of current venous access, use of a tunneled hemodialysis CVC as previous venous access, previous use of antimicrobials, and previous hospitalization (whether there had been a previous hospitalization or not).

**Table 3:** **Univariate and multivariate logistic regression analyses**

	**Simple logistic regression**		**Multiple logistic regression**	
	**OR (IC 95%)**	***p*** **-value**	**OR (IC 95%)**	***p*** **-value**
Diagnosis of hypertension	2.65 (1.16–6.03)	0.021		
Previous treatment (peritoneal dialysis)	2.45 (1.03–5.81)	0.042		
**Type of current venous access**				
CVC* vs. AVF**	10.72 (5.09–22.57)	<0.001	11.2 (5.17–24.29)	<0.001
**Duration of current venous access**				
0–30 vs. > 180 days	10.72 (2.95–38.96)	<0.001		
30–180 days vs. > 180 days	6.39 (2.64–15.45)	<0.001		
Use of previous venous access	3.01 (1.37–6.62)	0.006		
Tunneled CVC (previous access)	3.18 (1.09–9.28)	0.035		
Prior antimicrobial use	2.53 (1.21–5.28)	0.013		
Previous hospitalizations	5.90 (1.91–18.24)	0.002	6.63 (1.9–23.09)	0.003

*CVC: central venous catheter; **AVF: arteriovenous fistula.

With multiple logistic regression analysis, current access (CVC) and previous hospitalization were independent risk factors for the occurrence of BSI. Patients who had a CVC showed an 11.2-fold (CI 95%: 5.17–24.29) greater chance of developing BSI compared with patients who had an AVF. Previous hospitalization increased the chance of developing a BSI 6.6-fold (CI 95%: 1.9–23.09).

The most prevalent microorganisms isolated in blood cultures taken from the case group were Gram-positive bacteria (72.8%), followed by Gram-negative bacteria (25.9%), and fungi (1.2%). Among the Gram-positive microorganisms, *S. aureus* was isolated in 32.1% of cases, and of these, 38.5% showed resistance to *methicillin*. The second most frequent Gram-positive organism was *S. epidermidis* (13.6%), with 100% resistance to *methicillin*. Of the Gram-negative microorganisms isolated, 13.6% were of the enterobacteriaceae family and 12.3% were non-fermenters. For Gram-negative microorganisms, 33.3% were multi-resistant (Table 4).

**Table 4:** **Microorganisms isolated in the cases group, and profiles of resistance**

	**MR**	**S**	**Total**
**Gram-positives**	28 (47.5)	31 (52.5)	59 (72.8)
*Staphylococcus aureus*	10 (38.5)	16 (61.5)	26 (32.1)
*Staphylococcus epidermidis*	11 (100.0)	0 (-)	11 (13.6)
*Staphylococcus* coagulase negative non *S. epidermidis*	4 (57.1)	3 (42.9)	7 (8.6)
*Enterococcus faecalis*	1 (33.3)	2 (66.7)	3 (3.7)
Other Gram-positives*	3 (33.3)	9 (66.7)	12 (14.8)
**Gram-negatives**	7 (33.3)	14 (66.7)	21 (25.9)
Enterobacteriaceae**	4 (36.4)	7 (63.6)	11 (13.6)
Non-fermenters***	3 (30.0)	7 (70.0)	10 (12.3)
**Fungi**	0 (-)	1 (100.0)	1 (1.2)
*Candida albicans*	0 (-)	1 (100.0)	1 (1.2)
**Total of microorganisms**			81

Values are expressed as mean ± SD or *n* (%). MR: multi-resistant. S: sensitive. *Streptococcus acidominimus, *Streptococcus agalactiae*, *Streptococcus anginosus*, *Streptococcus bovis*, *Streptococcus pyogenes*, *Staphylococcus simulans*, *Staphylococcus capitis*, *Staphylococcus haemolyticus*, *Staphylococcus lugdunensis*; **Enterobacteriaceae: *Enterobacter spp*, *Enterobacter aerogenes*, *Enterobacter cloacae*, *Escherichia coli*, *Klebsiella pneumoniae*, *Serratia marcescens*, *Proteus mirabilis*; ***Non-fermenters: *Acinetobacter baumannii*, *Pseudomonas aeruginosa*, *Stenotrophomonas maltophilia*.

MRSA was isolated in 10 patients. Two of these patients had previous exposure to vancomycin and had a minimum inhibitory concentration to vancomycin of ≤0.05 μg/mL and 1.0 μg/mL.

With regard to progression for cases and controls, there were no significant differences in variables. Death occurred only in the cases group, retention in treatment was higher in controls, and shift to peritoneal dialysis showed a higher frequency in the cases group. Fifteen patients (46.7%) died from BSI, two (13.3%) from cardiovascular disease, and six (40%) from other causes, such as neoplasms, infections at other sites, and noninfectious complications at the vascular access. The median time to death after BSI was 4.6 ± 5.5 days (Table 5). Among the patients that died due to other causes, the death occurred from 45 to 672 days after the BSI.

**Table 5:** **Progression of the patients, cause of death and time of death post-BSI**

		**Cases (** ***n*** **= 81)**	**Controls (** ***n*** **= 81)**	***p*** **-value**
**Progression of the patients**				
Death		15 (18.5)	0 (-)	<0.001
Transfer of care		9 (11.1)	5 (6.2)	0.263
Duration of treatment		41 (50.6)	70 (86.4)	<0.001
Peritoneal dialysis		9 (11.1)	1 (1.2)	0.009
Transplant		7 (8.6)	5 (6.2)	0.549
**Cause of death in the cases**				
BSI		7 (46.7)		
Cardiovascular diseases		2 (13.3)		
Other*		6 (40.0)		
Total		15		
**Time of death post-BSI**				
		4.6 ± 5.5		

Values are expressed as mean ± SD or *n* (%). *neoplasms, infections at other sites, cardiovascular diseases, and noninfectious complications in the vascular access.

## Discussion

For patients undergoing renal replacement therapy, infection is the leading cause of morbidity and the second leading cause of mortality [1]. In Brazil, it is estimated that 97,586 patients currently receive chronic dialysis treatment [26]. According to North American data, the rates of BSI among hemodialysis patients range between 0.5 and 27.1 per 100 patients/month depending on the type of venous access used [2]. Results of a Brazilian study reported that rates varied between 0.55 and 7.32 per 1000 venous accesses/day [27].

In the present study, risk factors for BSI were evaluated in patients on hemodialysis at a hemodialysis service in São Paulo, Brazil. The study was a case–control design, which can analyze risk factors related to a determined condition. Patients presenting with BSI during the study period were included as cases, and controls were those patients who did not develop the studied event (pairing criteria: age, previous time on hemodialysis, and diagnosis of diabetes mellitus).

Use of a CVC compared with use of an AVF, and previous hospitalization were independent risk factors for the occurrence of BSI among patients on hemodialysis treatment. Use of a CVC was associated with an increased risk of developing BSI (OR: 11.2; CI 95%: 5.17–24.29; p < 0.001) compared with the use of an AVF. This finding aligns with previous trials of antimicrobial locks, which reported a higher occurrence of BSI among hemodialysis patients with CVC (3-4 cases per 1000 catheter-days) [28]. In a longitudinal cohort study, Xui *et al.* found that the rates of BSI in patients using CVC were three times higher than in patients using AVF (p < 0.001) [8]. A case–control study conducted at a university hospital in Greece demonstrated, through multivariate logistic regression, that patients using CVC had a higher risk of BSI compared with patients using AVF (OR: 2.93; p = 0.047) [10].

In the current study, only five patients (four cases and one control) used a non-tunneled hemodialysis CVC. In these patients, three had started hemodialysis within 60 days and were awaiting the manufacture of an AVF, and the other two had been undergoing treatment for a longer period of time and had spent longer with a non-tunneled hemodialysis CVC because of difficulties in the insertion of a tunneled hemodialysis CVC or the manufacture of an AVF.

Previous hospitalization increased the risk of BSI 5.33-fold (p = 0.003). There are few reports in the literature on the association between BSI and previous hospitalizations in patients undergoing hemodialysis treatment. Nguyen *et al*. in a study performed between 2005 and 2011, evaluated BSI from MRSA in patients on dialysis treatment, and found that 70% of patients who developed BSI were hospitalized at least once in the year prior to the infection [6]. In a study by Barbosa *et al*. the researchers reported that, in 320 chronic renal patients, previous hospitalization was an important risk factor for colonization of multi-resistant microorganisms (e.g., VRE) [29]. And Aktas *et al*. in a study of 70 patients on dialysis treatment, reported a higher colonization for MRSA in patients who had been hospitalized within the previous 6 months compared with those who had not [30].

In univariate logistic regression analysis, systemic arterial hypertension, peritoneal dialysis as a prior treatment, duration of venous access, presence of previous venous access, use of tunneled hemodialysis CVC as a previous access, and previous antimicrobials were associated with a higher occurrence of BSI. No data was found in the literature on the possible relationship between systemic arterial hypertension and previous peritoneal dialysis with the occurrence of BSI in this population.

For examination of time of duration of current venous access as a risk factor, BSI was found to be associated with access that had been gained within 30 days. Duration of current venous access was analyzed using three categories (0–30 days, 30–180 days, and >180 days). BSI was higher in those who had had the CVC inserted within 30 days, which underscores the need for greater care during the CVC insertion procedure. Napalkov *et al*. in a study that evaluated infectious and noninfectious complications in CVC, reported that the majority of BSI occurred within the first 90 days, with an incidence rate/1000 catheter-days of 5.1 (CI 95%: 3.7–4.3) [31]. There was also a higher risk of infection for catheters inserted within the 6 months following infection, reinforcing the need for care during this period.

The use of previous venous access and, primarily, previous access of tunneled hemodialysis CVC, was associated with a higher occurrence of BSI. As mentioned above, studies have reported that CVC use is associated with a higher incidence of BSI compared with the use of an AVF [8-10]. References to previous venous access as a risk factor for BSI were not found in the literature.

Previous use of antimicrobials was associated with a higher occurrence of BSI (OR: 2.53; p = 0.013). As with other variables, data were collected on the use of antimicrobials within 6 months before the event. We found no specific studies that related prior antimicrobial exposure to BSI in patients undergoing hemodialysis treatment. However, Lim *et al*. in a case–control study conducted with patients seen in the emergency department of a tertiary hospital in Australia, showed through multivariate analysis that prior antimicrobial use increased the risk of developing BSI by multi-resistant microorganisms (OR: 5.49; p < 0.001) [32]. According to the Centers for Disease Control and Prevention, the rational use of antimicrobials is an important measure for controlling the spread of multi-resistant microorganisms [23].

Gram-positive microorganisms were the most prevalent (72.8%) found in the current study. Among them, *S. aureus* was the most frequently isolated (32.1%), with about 38.5% resistant to oxacillin. Other studies have also reported a high prevalence of Gram-positive microorganisms in patients undergoing hemodialysis treatment, primarily *S. aureus* [7,10,11]. Despite the high prevalence of Gram-positive microorganisms observed in the current study, Gram-negative microorganisms accounted for 25.9% of isolates in blood cultures. In a university hospital in Spain, microorganisms isolated in blood cultures from patients with kidney disease were: Gram-negative bacteria, 52.3%; Gram-positive bacteria, 46.5%; and fungi, 1.2%. *Escherichia coli* was the most frequent microorganism (27%) [33].

With regard to resistance profiles, microorganisms such as MRSA and VRE have been isolated in surveillance cultures collected from patients on dialysis treatment [29,34,35]. Aktas *et al*. used molecular typing to show a high similarity between MRSA strains isolated from surveillance cultures and clinical cultures collected from patients on dialysis treatment [30]. And a study conducted at long-term care facilities in Hong Kong reported a significant dissemination of MRSA, reinforcing the need to adopt measures to reduce the transmission of this microorganism [36].

The independent risk factors for BSI identified in the current study were use of a CVC compared with use of an AVF, and previous hospitalization. Although previous studies have indicated a relationship between the presence of CVC and BSI, our study expands the characteristics of this group, highlighting the importance of care during the initial period and the period of up to 6 months of catheter duration. Furthermore, the study highlights the importance of previous hospitalization as a risk factor for BSI. To the best of the authors’ knowledge, this factor has not been reported in previous studies and requires further research.

This study had some limitations inherent in the retrospective character of the data collection. However, patient records at the study institution were completed with significant caution, given the characteristics of a service dedicated to the treatment of chronic renal patients, and followed the high standards of the organization. Additionally, as there are standardized protocols for performing blood cultures for these patients, it is unlikely that events were underdiagnosed in the study period.

Preventative measures against BSI related to CVC should be strengthened and effectively applied to hemodialysis treatment units, as well as reducing the use of this device whenever possible, prioritizing the use of AVF. Considering that previous hospitalization was an independent risk factor for BSI, patients who developed BSI after hospitalization are probably infected by microorganisms during this period. Standard precautions should be enforced to prevent microorganism dissemination during hospitalization.

A reduction in the number of infections in this population may contribute to a decrease in hospital admissions, since infections are a leading cause of hospitalization in patients on hemodialysis treatment.

## Conclusions

The risk factors for BSI in chronic renal disease patients receiving hemodialysis were previous hospitalization and use of a CVC. The most prevalent microorganisms were Gram-positive bacteria, of which 47.5% were multi-resistant. The results of this study suggest the need to intensify measures to prevent and control BSI in hemodialysis patients, among which are specific measures for insertion and maintenance of the CVC, and the judicious use of this device. Efforts should be made to reduce exposure of these patients to hospitalization to minimize the risk of BSI. Future research could evaluate the impact of surveillance cultures to prevent microorganism dissemination in this population.

### Consent

Written informed consent was obtained from the patient for the publication of this report and any accompanying images.
